# Troubleshooting: intraperitoneal migration of a dedicated plastic stent during endoscopic ultrasound-guided hepaticogastrostomy

**DOI:** 10.1055/a-2791-4644

**Published:** 2026-02-17

**Authors:** Hirotsugu Maruyama, Yuki Ishikawa-Kakiya, Yuji Kawata, Tatsuya Kurokawa, Yoshinori Shimamoto, Kojiro Tanoue, Yasuhiro Fujiwara

**Affiliations:** 1 12936 Department of Gastroenterology, Graduate School of Medicine, Osaka Metropolitan University, Osaka, Japan

Complications of endoscopic ultrasound-guided hepaticogastrostomy (EUS-HGS) have decreased with improved techniques and devices, with cholangitis and bleeding now being the most common 1 . Although fatal stent migration was previously a major concern, anti-migration stents have made it rare 2 3 . Nevertheless, severe complications can still occur due to endoscopist inattention or misjudgment.

In this case, a dedicated plastic stent migrated intraperitoneally because the placement relied solely on fluoroscopy alone. An additional EUS-HGS via B3 was performed, allowing the successful retrieval of the migrated stent through the new tract.

**Fig. 1:**
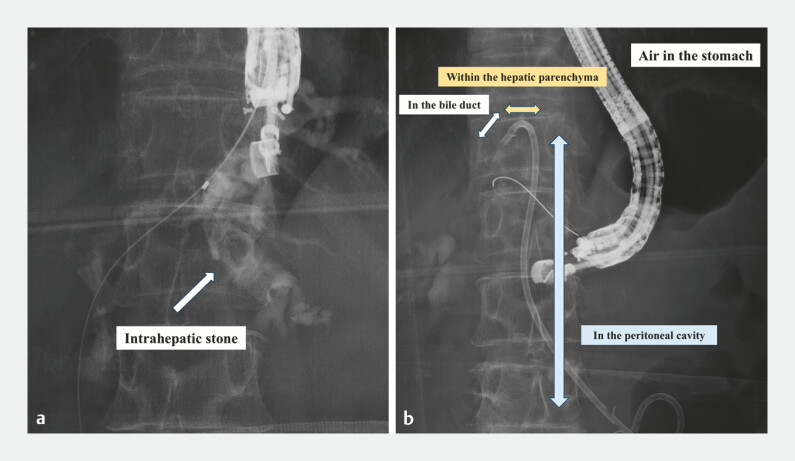
A fluoroscopic image of endoscopic ultrasound-guided hepaticogastrostomy. **a** A fluoroscopic image of cholangiography showing intrahepatic stones. **b** A fluoroscopic image after the migration of the dedicated plastic stent into the peritoneal cavity.

**Fig. 2:**
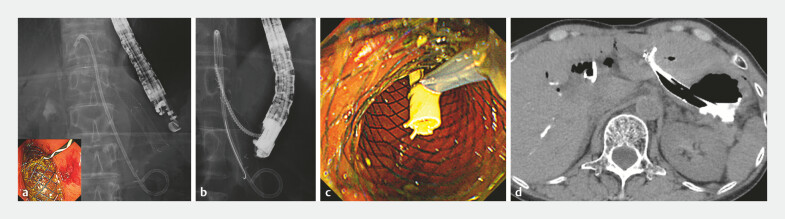
The clinical course after the migration of the dedicated plastic stent. **a** Endoscopic ultrasound-guided hepaticogastrostomy was performed at B3, and a fully covered metal stent was placed. **b** A fluoroscopic image showing the insertion of the drill dilator into the stent lumen. **c** After pulling the stent proximally toward the endoscope using the drill dilator, it was grasped with a snare. **d** A post-endoscopic procedural CT image. No obvious bile and contrast leakage were observed. CT, computed tomography.

A 66-year-old woman with prior gastrojejunostomy for duodenal obstruction due to cholecystitis was referred for cholangitis caused by intrahepatic stones. EUS-HGS was attempted by placing a dedicated plastic stent (PS). However, the stones prevented deep stent advancement, and it was inserted using fluoroscopic guidance alone. The stent was not seen endoscopically, leading us to conclude that it had been inadvertently deployed into the peritoneal cavity ( Fig. 1 ). We then punctured B3 and placed a metal stent (MS) to create a wider drainage tract and reduce intraperitoneal leakage. Through the MS tract, we attempted to locate the PS fluoroscopically but could not identify it. Using a 22-gauge needle and a 0.018-inch guidewire, we accessed the PS in the hepatic parenchyma, but could not direct the guidewire toward the MS tract. Finally, we succeeded in advancing a GW into the lumen of the migrated PS via the MS route. Using a drill dilator, we were able to retrieve the stent into the stomach ( Video 1 ). The postoperative course was uneventful ( Fig. 2 ).

Troubleshooting: intraperitoneal migration of a dedicated plastic stent during endoscopic ultrasound-guided hepaticogastrostomy.Video 1

This case serves as an important reminder that endoscopic and fluoroscopic imaging must be fully utilized. It also presents a method for retrieving a stent even if it migrates into the abdominal cavity, making it a very rare and valuable report.

Endoscopy_UCTN_Code_TTT_1AS_2AH
